# Angioedema: Clinical and Etiological Aspects

**DOI:** 10.1155/2007/26438

**Published:** 2007-11-28

**Authors:** Kanokvalai Kulthanan, Sukhum Jiamton, Kanonrat Boochangkool, Kowit Jongjarearnprasert

**Affiliations:** ^1^Department of Dermatology, Faculty of Medicine Siriraj Hospital, Mahidol University, 2 Prannok Road, Bangkoknoi, Bangkok 10700, Thailand; ^2^Department of Pharmacy, Faculty of Medicine Siriraj Hospital, Mahidol University, 2 Prannok Road, Bangkoknoi, Bangkok 10700, Thailand

## Abstract

Angioedema is an abrupt swelling of the skin, mucous membrane, or both including respiratory and gastrointestinal tracts. This study aimed to report an experience of angioedema in a university hospital with respect to etiologies, clinical features, treatment, and outcome. One hundred and five patients were enrolled. About half had angioedema without urticaria. The common sites of involvement were periorbital area and lips. Forty five patients (49%) had systemic symptoms. The most common cause of angioedema was allergic angioedema. Nonsteroidal anti-inflammatory drug-induced angioedema and idiopathic angioedema were detected in 20% and 18%, respectively. Among patients with allergic angioedema, 41.7% were caused by food, 39.6% by drugs. Thirty seven patients (39%) had recurrent attacks of angioedema. Mean standard deviation (SD) number of attacks in patients with recurrent angioedema was 3.9 (2.7) (ranging from 2 to 10 times). Patients who had older age and multiple sites of skin involvement had tendency to have systemic symptoms.

## 1. INTRODUCTION

Angioedema is an abrupt swelling of the skin, mucous membrane, or both including respiratory and gastrointestinal tracts [1]. The swelling is nonpitting, skin-colored, or sometimes erythematous, and shows a predilection for areas where the skin is lax. Swellings normally subside in around 24 hours or more and resolve without discoloring the skin. Angioedema is a consequence of local increase in permeability of subcutaneous or submucosal capillaries and postcapillary venules causing local plasma extravasation in response to mediators such as histamine, bradykinin [2]. Angioedema and urticaria may coexist. Allergic angioedema (histamine-induced angioedema) is a hypersensitivity reaction to various causes such as drugs, foods, insect venoms [1]. Kinin-induced angioedema is believed to be caused by bradykinin-induced activation of endothelial cells resulting in vasodilatation and capillary leakage [3]. Two different types of kinin-induced angioedema are known, hereditary and drug-induced forms [2]. However, idiopathic forms with unknown cause and mixed forms also exist [2].

Greaves et al. classified angioedema according to the causes, that is, allergic, nonsteroidal antiinflammatory drug (NSAID)-induced, idiopathic angioedema, angioedema associated with idiopathic or autoimmune urticaria, associated with urticarial vasculitis, infection, and infestations, angioedema with eosinophilia, associated with some physical urticarias and with cholinergic urticaria, associated with allergic contact urticaria, defect in the plasma inhibitor of the first component of complement (C1-INH deficiency)-hereditary (HAE), acquired angioedema, HAE with normal C1-INH in women, and angiotensin-converting enzyme inhibitor (ACEI)-induced angioedema [4].

There were some reports of immunogenetic differences between Oriental and Caucasian populations, resulting in differences in the clinical presentation and frequency of autoantibodies detected in some autoimmune diseases [5, 6]. Moreover, different dietary habits between Asian and Western countries as well as the socioeconomic status may cause different allergen sensitization. Most of the studies about angioedema were reported from Western countries. It is interesting to know the prevalence and clinical features of Asian patients with angioedema. This study aims to report an experience of angioedema in a university hospital with respect to etiologies, clinical features, treatment, and outcome.

## 2. MATERIALS AND METHODS

This study has been approved by the Siriraj Hospital institutional review board. Records of patients with angioedema who attended the outpatient department of Siriraj Hospital between January 2005 and December 2006 were retrospectively reviewed. Patients aged at least 15 years were assigned a diagnosis of angioedema by an attending physician by having an abrupt and short-lived nonpitting swelling of skin in areas such as the face, lips, oral cavity; mucous membranes (including the respiratory and intestinal tract) or both, without evidence of infectious, traumatic, or other clear causes of the swelling.

Demographic data, etiologies, clinical features, course of the disease, treatment, and outcome were studied. With the exception of ACEI, a drug reaction was regarded as a likely cause if taken within 24 hours before onset of angioedema. All suspected drugs used were discontinued or replaced with chemically unrelated drugs. Food intolerance was regarded as a likely cause if angioedema occurred within 2 hours after ingestion of suspected foods. Skin prick testing, drug provocation tests, and oral food challenge tests were performed if necessary and if the disease severity was not severe. Foods and drugs that were subsequently tolerated after challenging in remission period were excluded from the causes of angioedema. Laboratory investigation included complete blood counts, urinalysis, erythrocyte sedimentary rate (ESR), stool examination, and other investigations that were necessary for the individuals, that is, BUN, creatinine, AST, ALT, ALP, bilirubins, total protein, albumin, HbsAg, anti-HCV, free T3, free T4, thyroid stimulating hormone (TSH), antithyroid autoantibodies, that is, antithyroglobulin, and antimicrosomal antibodies (passive hemagglutination test; Hausen Bernstein CO., LTD, 12076 Santa Fe Drive, Lenexa, KS 66215, USA), antinuclear antibodies (ANA), cryoglobulins, serum complement level (C4 determined by Nephelometry; Beckman, Gagny, France; C1q measured by single radial immunodiffusion using monospecific antisera), chest, and sinus X-ray studies. Other data obtained were personal history of allergic rhinitis, asthma, atopic dermatitis, and allergic conjunctivitis.

## 3. STATISTICAL METHODS

All statistical analyses were two sided at 95% confidence level using SPSS version 10 for Windows. The t-test and Chi-squared test was, respectively, used for statistical analysis among continuous and categorical data.

## 4. RESULTS

One hundred and five patients were enrolled in the study and 82 cases (78.1%) were female. Mean (SD) age of the patients was 39.4 (18.4) years with an age ranged of 15 to 88 years. Fifty five patients (52.4%) had angioedema without urticaria (Table 1). Forty five patients (49%) had systemic symptoms. Average duration of individual lesion of angioedema was 41.9 hours with a range of 24 to 168 hours. Two thirds of the patients were presented with the first episode. Mean age of the first attack was 38.2 years. One fourth of the patients had personal history of atopy, the most common of which was allergic rhinitis.

Patients with systemic symptoms had statistically significant higher mean age (44.2 versus 35.8 years, P=.032) and multiple sites of angioedema (P=.001) than those without systemic symptoms.

Table 2 shows signs and symptoms in various groups of angioedema. Two thirds of the patients stated itching as a symptom. However, the group of angioedema with urticaria had statistically significant higher proportion of itchy sensation than those without urticaria (P<.001). Among cases who had systemic symptoms, the common sign and symptom were chest discomfort and/or dyspnea. Only 9 of 105 patients (8.6%) had hypotension. Seven of these 9 patients had the first attack of angioedema. Five of them had angioedema with urticaria. All of them also had other systemic symptoms. Concerning the causes, 2 cases had angioedema from insect stings, 2 cases from food reactions, 1 case from NSAID, 1 case from amoxicillin, the others from unspecified causes.

Table 3 shows anatomical sites of involvement of angioedema. Overall, the common sites of involvement were periorbital area and lips. There were no statistically significant differences in anatomical site involvement between the group with or without urticaria. In this study, only one patient had angioedema on her lips caused by ACEI (perindopril). Patients with NSAID-induced angioedema had periorbital swelling in 95.2%; lip swelling, 14.3%; tongue swelling, 4.8%; and swelling of extremities, 4.8%, and tended to have higher percentage of periorbital swelling compared with allergic angioedema group (95.2% versus 68.8%, P<.05). The group with allergic angioedema was more likely to have lip swelling when compared with the group with NSAID-induced angioedema (43.8% versus 14.3%, P<.05) (data not shown).

Table 4 shows presumed causes of angioedema in this study. The most common cause was allergic angioedema. Nonsteroidal anti-inflammatory drug- (NSAID-) induced angioedema and idiopathic angioedema were detected in 20% and 18%, respectively. NSAIDs responsible for angioedema in our study were ibuprofen (57%), aspirin (19%), diclofenac (9.5%), mefenamic acid (4.8%), naproxen (4.8%), and meloxicam (4.8%). Among 48 patients with allergic angioedema, 41.7% cases were caused by food, 39.6% by drugs, 4 patients (8.3%) by insects (2 cases by fireant, 2 cases by unknown insects), and 5 patients (10.4%) were affected by aeroallergens. The most common food that induced angioedema was seafood (70%). The common drugs that presumed to be the causes of angioedema were antibiotics (12 out of 19 cases; 63.2%), the most common was amoxicillin (3 in 12 cases; 25%).

Among 48 patients with allergic angioedema, personal history of atopy was found in 12 patients (25%) (allergic rhinitis 10 cases, 20.8%; asthma 4 cases, 8.3%). Seven of 21 patients (33.3%) with NSAID-induced angioedema had atopic histories (allergic rhinitis 4 cases, 19%; asthma 6 cases, 28.5%). None in idiopathic angioedema group had history of atopy. Six of 20 patients (30%) who had reactions to foods had atopic histories (allergic rhinitis 5 cases, 25%; asthma 1 case, 5%).

Acquired C1-INH deficiency was presumed to be the cause of angioedema in 4 patients (3.8%) who were diagnosed and fulfill criteria of systemic lupus erythematosus (SLE). All patients had decreased level of C4 and C1q and had no other explanations for the causes of their angioedema. However, it was not confirmed by special serologic testing for C1-INH levels. Among patients who had angioedema associated with contact urticaria, the responsible agents were hair dye, face cleansing foam, herb, and corrosive agent used in tin cleansing. Two patients had upper respiratory tract infection simultaneously with angioedema.

With exception of patients with C1-INH deficiency, 90 patients (90%) were treated with antihistamine. Other medications included corticosteroids (71%), adrenaline (24%), H2 blocker (20%), and proton pump inhibitor (3%). Fifteen percent of the patients received intravenous fluid replacement and thirteen percent received oxygen therapy. However, no patients needed emergency intubation. Drugs suspected to be the cause of angioedema had been discontinued in all cases. Patients with acquired C1-INH deficiency received prednisolone treatment for the underlying LE disease as well as prophylactic androgenic compounds. One patient with HAE received danazol as a prophylactic medication with a good response. He had a previous history of emergency tracheostomy in the rural hospital.

Two thirds of the patients had their first visit at the emergency department. However, only three percent of the patients were admitted as inpatient. No patients in the present study died as a result of angioedema. Thirty seven patients (39%) had recurrent attacks of angioedema. Mean (SD) number of attacks in patients with recurrent angioedema was 3.9 (2.7) (ranging from 2–10 times).

## 5. DISCUSSION

Most patients with angioedema in this study were female. This is similar to the study of Cohen et al. which 63% of their patients were female [7]. However, male predominance has also been reported [8, 9].

In 2002, Vichyanond et al. reported the prevalence of allergic rhinitis; and asthma in Thai university students was 26.3% and 8.8%, respectively, [10]. In the present study, the personal history of allergic rhinitis and asthma in patients with angioedema were 19.2% and 13.1%, respectively. When compared with the prevalence from Vichyanond et al., there were no statistically significant increases in the prevalence of allergic rhinitis and asthma in our patients with angioedema and various subgroups of angioedema (allergic angioedema, idiopathic and food-induced) except for NSAID-induced angioedema group which had statistically significant higher prevalence of asthma (P=.012).

Acute allergic angioedema is usually accompanied by urticaria [1]. Allergic reactions to food, drugs, environmental contacts, insect stings, and other substances can cause IgE-mediated immediate hypersensitivity reaction. Systemic release of histamine and other mediators from mast cells may cause angioedema, either isolated or as a part of systemic anaphylaxis. It usually occurs within 1 to 2 hours of exposure to the responsible allergen. Its onset is reliant on prior sensitizing exposure. Allergic angioedema is more common in patients with atopy, that is, allergic rhinitis, asthma, and atopic dermatitis. However, allergic reactions to foods or medications may be seen in the absence of diseases associated with atopy; and some medications such as radiocontrast media and opiates can cause nonimmunologic mast cell degranulation. Similar to acute urticaria, diagnosis of the cause of angioedema depends principally on the history. Most patients are fully aware of the association of allergens or drugs with the onset of acute angioedema. Skin prick testing and radioallergosorbent serum testing, which demonstrate the specific IgE-mediated hypersensitivity, may provide supportive information in some instances [1].

In our study, 21 of 48 patients (43.8%) with allergic angioedema had urticarial wheals accompanied with angioedema. About half of patients who had personal histories of atopy had allergic angioedema. The common causes of allergic angioedema in this study were foods (mostly seafood), and drugs (other than NSAIDs). It should be noted that penicillin is less commonly used in our country nowadays.

IgE- and mast cell-mediated reactions leading to urticaria and angioedema can be caused by physical stimuli such as cold urticaria. In this study, besides contact urticaria and angioedema, no patients had symptoms caused by other physical stimuli.

Because of the increasing of ACEI use in this era, many studies reported an increase in ACEI-induced angioedema which had been reported to occur in 0.1–0.5% of patients taking these drugs [11]. Megerian et al. reported that between 1985 and 1990, 35% of patients presenting with angioedema were using ACEI [12]. Pigman et al. reported that 24% of angioedema cases presenting to an emergency department between 1984 and 1991 were associated with ACEI use [8]. Cohen et al. reported a 5-year study conducted in a large, urban, university hospital between 1994 and 1998 [7]. Fifty eight percent of their patients with angioedema were using ACEIs. In spite of the popular usage of ACEIs in our country, only one patient (1%) in the present study had angioedema associated with ACEI usage. Similarly, Thai FDA report states that out of 364 cases of drug-induced angioedema in 2006, 101 patients (27.7%) had NSAID-induced angioedema (mostly ibuprofen, 29.7%; aspirin, 10%) and 7 patients (1.9%) had ACEI-induced angioedema. A 4-fold increase in the risk of ACEI-induced angioedema had been reported in Black-American patients, suggesting the result of differences in the kallikrein-kinin system with increased sensitivity to bradykinin [13]. Perhaps, this racial difference may explain a few cases of ACEI-induced angioedema in the present study.

In our study, NSAIDs were the second common cause of angioedema. The most common NSAID causing angioedema in this study was ibuprofen. The prevalence of urticaria and angioedema to NSAIDs in the general populationhas been reported to be 0.1–0.3% [14, 15]. However, in selected atopic populations, there were some reports of increases in frequency from childhood to young adulthood [16, 17]. A variety of NSAIDs can cause angioedema and aspirin reported to be the most common [1]. True allergic reactions to NSAIDs are rare. Reactions to these drugs are often termed pseudoallergic reaction explained by pharmacologic properties of the drugs that inhibit COX enzymes. Quiralte et al. reported that that the most frequent NSAID adverse reaction observed in the skin is facial angioedema [18].

It has also been recognized that NSAIDs can aggravate skin lesions in up to one third of patients with active chronic urticaria, but they do not appear to induce any eruptions during the symptomless periods of the disease [16]. Challenges to confirm the patient’s hypersensitivity to NSAIDs identified as probable causes of previous reactions are deferred, in order to avoid provoking unnecessary risks. A patient with an adverse NSAID reaction should ideally have an alternative NSAID if necessary.

In our study, patients with NSAID-induced angioedema had statistically significant increased prevalence of asthma but had no increased prevalence of allergic rhinitis and atopic dermatitis. All patients with NSAID-induced angioedema had facial involvement and the most common was periorbital area. The group with NSAID-induced angioedema showed statistically significant periorbital involvement (P=.001) when compared with other subgroups of angioedema.

Patients with serum sickness or necrotizing vasculitis, angioedema, and urticaria are mediated by immune complex-mediated reaction [7]. In our study, only 2 patients (1.9%) had angioedema associated with urticarial vasculitis.

The mainstay of emergency medical treatment of HAE is intravenous fresh frozen plasma or C1 inhibitor concentrate or lyophilized vapor-heated C1 inhibitor concentrate [19, 20]. For patients in whom attacks are infrequent and not life-threatening, the treatment may be restricted to avoidance of provocative factors, that is, trauma, estrogens, and ACEI administration [1]. However, exacerbation of HAE can be prevented by aminocaproic acid, tranexamic acid, or anabolic steroids such as danazol and stanozolol [21, 22]. Treatment of acquired C1-INH deficiency requires treatment of underlying disease plus treatment with afore-mentioned drugs as that for the treatment of HAE.

Severe symptoms of other forms of angioedema may be treated with epinephrine injection and less severe symptoms are often controlled by antihistamine. The use of corticosteroids may reduce the possibility of relapse [1].

In summary, our study provided an overview of angioedema based on etiological aspects and clinical features. Patients who had older age and multiple sites of skin involvement had tendency to have systemic symptoms. In contrast to reports from Western countries, ACEI-induced angioedema was uncommonly detected in the present study. Ibuprofen was the most common NSAID that caused angioedema in this study whereas aspirin was reported to be the most common culprit NSAID in Western countries.

## Figures and Tables

**Table 1:** Demographic data in various group of angioedema.

Characteristics	Angioedema total (n=105)	Angioedema without urticaria (n=55)	Angioedema with urticaria (n=50)	Angioedema with systemic symptoms (n=45)
*Sex*: Number (%)				
Male	23(21.9)	13(23.6)	10 (20)	9 (20)
Female	82(78.1)	42(76.4)	40 (80)	36 (80)
*Age*, years: mean (SD)	39.4(18.4)	40.5(18.5)	38.3 (18.3)	44.2 (20.9)*
*Duration of individual lesion*,hours: mean (SD)	41.9(40.7)	43.5(40)	39.8 (43.2)	45.9 (47.6)
*Age of onset of first attack*,years: mean (SD)	38.2(18.5)	39.2(19.4)	37.3 (17.7)	42.4 (21.7)
*Number of attack*:mean (SD)	3.9(2.7)	3.9(2.6)	3.7 (3.2)	3.3 (2.1)
*Personal atopic history*:number (%)	25(25.3)	17(33.3)	8 (16.7)	13 (30.2)
Allergic rhinitis	19(19.2)	14(27.5)	5 (10.4)	9 (20.9)
Allergic conjunctivitis	4(4)	3(5.9)	1 (2.1)	1 (2.3)
Asthma	13(13.1)	7(13.7)	6 (12.5)	7 (16.3)
Atopic dermatitis	1(1)	0	1 (2.1)	1 (2.3)

*P<.05.

**Table 2:** Signs and symptoms associated with angioedema.

Symptoms,number (%)	Angioedema total (n=105)	Angioedema without urticaria (n=55)	Angioedema with urticaria (n=50)	Angioedema with systemic symptoms (n=45)
*Urticaria*	50(47.6)	0	50 (100)	17 (37.8)
*Itchy skin*	62(59)	18(32.7)*	44 (88)	25 (55.6)
*Pain*	7(6.7)	3(5.5)	4 (8)	4 (8.9)
*Stuffed nose*	8(7.6)	5(9.1)	3 (6)	6 (13.3)
*Systemic symptoms*	45(42.9)	28(50.9)	17 (34)	45 (100)
Fainting	6(5.7)	1(1.8)	5 (10)	6 (13.3)
Unconciousness	1(1)	0	1 (2)	1 (2.2)
Dyspnea/wheeze	42(40)	26(47.3)	16 (32)	42 (93.3)
Abdominal pain	4(3.8)	2(3.6)	2 (4)	4 (8.9)
Hypotension	9(8.6)	4(7.3)	5(10)	9(20)

*P<.001.

**Table 3:** Anatomicsites of involvement of angioedema.

Site of edema, number (%)	Angioedema total (n=105)	Angioedema without urticaria (n=55)	Angioedema with urticaria (n=50)	Angioedema with systemic symptoms (n=45)
*Face*	101(96.2)	53(96.4)	48 (96)	45 (100)
Periorbital	70(66.7)	41(74.5)	29 (58)	37 (82.2)
Lip	43(41)	19(34.5)	24 (48)	21 (46.7)
*Tongue *	3(2.9)	2(3.6)	1 (2)	1 (2.2)
*Glottis*	7(6.7)	4(7.3)	3 (6)	7 (15.6)
*Genitalia*	1(1)	1(1)	0 (0)	0 (0)
*Extremities*	11(10.5)	3(5.5)	8 (16)	5 (11.1)
*Multiple sites*	31(29.5)	14(25.5)	17 (34)	22 (48.9)

**Table 4:** Presumed causes of angioedema in 105 patients in this study.

Causes	Number (%)
Allergic angioedema*	48(45.7)
NSAID-inducedangioedema	21(20)
Idiopathicangioedema	19(18.1)
Associated with contacturticaria	4(3.8)
Acquired C'1 INHdeficiency	4(3.8)
Associated with chronicidiopathic or autoimmune chronic urticaria	3(2.9)
Associated with urticarial vasculitis	2(1.9)
Infection andinfestations	2(1.9)
Angiotensin-convertingenzyme inhibitor-induced angioedema	1(1)
Hereditaryangioedema (C'1 INH deficiency)	1(1)
Associated with physicalurticarias	0

*Angioedema occurring within 1 to 2 hours of exposure to the offending allergen, caused by immediate type hypersensitivity reaction.
